# Supplementary material for: Optimization of differentiation and transcriptomic profile of THP-1 cells into macrophage by PMA
Source: PLoS One. 2023 Jul 17;18(7):e0286056. doi: 10.1371/journal.pone.0286056 (PMC10351730; doi:10.1371/journal.pone.0286056)
Supplement: S1 File — (ZIP) [file pone.0286056.s001.zip › supporting files/RNA-seq report.zip!/RNA-seq report/2_GeneQuantification_result/fpkm.summary.pdf]

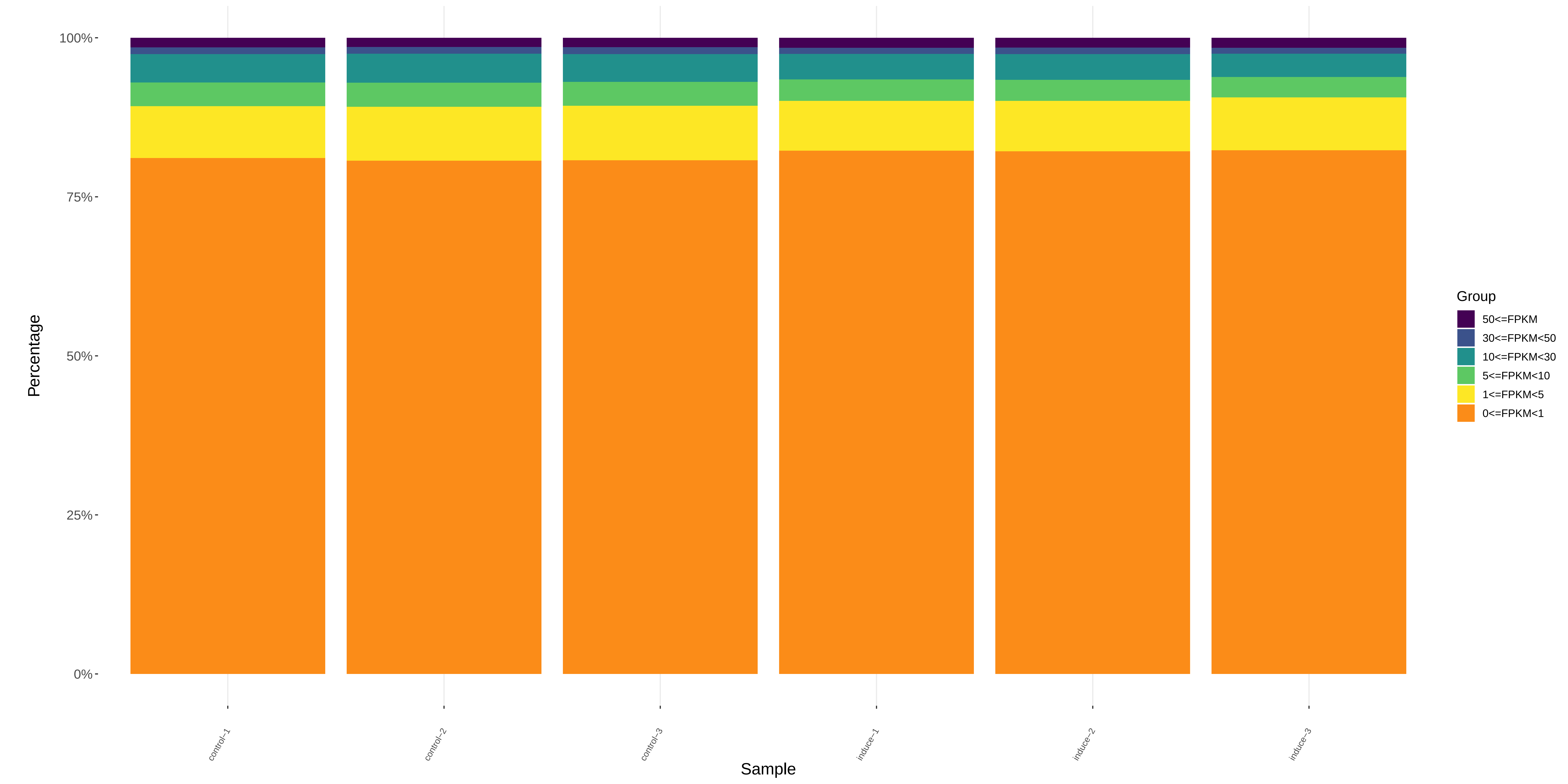
100%
75%
50%
25%
0%
Percentage
control−1
control−2
control−3
induce−1
induce−2
induce−3
Sample
Group
50<=FPKM
30<=FPKM<50
10<=FPKM<30
5<=FPKM<10
1<=FPKM<5
0<=FPKM<1
